# Supplementary material for: Heterogeneous entity representation for medicinal synergy prediction
Source: Bioinformatics. 2025 Jan 15;41(1):btae750. doi: 10.1093/bioinformatics/btae750 (PMC11745903; doi:10.1093/bioinformatics/btae750)
Supplement: btae750_Supplementary_Data [file btae750_supplementary_data.zip › c6dce_supplement.pdf]

# Supplementary Materials for "Heterogeneous Entity Representation for Medicinal Synergy Prediction"

Jiawei Wu, Jun Wen, Mingyuan Yuan, Anqi Dong, Shuai Gao, Ren Wang, and Can Chen

To further validate our approach, we conducted supplementary experiments using additional gene expression data from the Cancer Cell Line Encyclopedia (CCLE) and Genomics of Drug Sensitivity in Cancer (GDSC) datasets, in place of the COSMIC dataset used in the main experiments. Below, we outline the data processing steps, experimental setup, and results for each dataset.

## CCLE Dataset

For the CCLE dataset, we replaced COSMIC's gene expression data with gene expression profiles from CCLE to investigate the generalizability of our model across different data sources. Most cell lines in our O'Neil and ALMANAC datasets have matching identifiers in CCLE, allowing for a direct comparison. However, two cell lines out of 32 in the O'Neil dataset and ten out of 55 in the ALMANAC dataset did not have corresponding gene expression data in CCLE, which were available in COSMIC. To address this, we established two models for fair comparison:

- **Overlap-COSMIC Model**: A model trained using only the cell lines that are common between COSMIC and CCLE, leveraging COSMIC's gene expression data.
- **Overlap-CCLE Model**: A counterpart model trained with the same overlapping cell lines but using CCLE gene expression data.

In the CCLE dataset, each cell line is associated with 57,820 gene expression features. To streamline the model and reduce computational complexity, we performed feature selection, retaining approximately 2,048 genes for further experiments.

## GDSC Dataset

In addition to CCLE, we employed the GDSC dataset, which includes two versions: GDSC1 and GDSC2. Unlike COSMIC and CCLE, GDSC does not provide explicit gene expression data for cell lines. Instead, GDSC records experimental results for pairs of cell lines and drugs across multiple conditions. Each row in GDSC contains the following eight attributes: *cell_line*, *drug_id*, *min_conc*, *max_conc*, *LN_IC50*, *AUC*, *RMSE*, and *Z_SCORE*. The first four attributes specify the experimental setup—linking a cell line to a drug along with its minimum and maximum concentrations—while the latter four capture the experimental outcomes: the log-transformed half-maximal inhibitory concentration (LN_IC50), the area under the dose-response curve (AUC), root mean square error (RMSE), and drug response Z-score. To integrate GDSC data, we standardized each of these four experimental metrics, making them suitable for model training and evaluation. This approach allows us to assess the robustness of our method when different pharmacogenomic features are used in place of gene expression profiles.

## Experimental Description

Our supplementary experiments with CCLE and GDSC involved training and evaluating models as detailed in the main manuscript. The comparative analysis between the overlap-COSMIC and overlap-CCLE models, as well as the integration of standardized GDSC metrics, highlights the adaptability of our framework to diverse data sources. We present these results in Table 1 to provide a comprehensive assessment of HERMES's performance and the effect of alternative datasets on drug synergy prediction.

Table 1: Performance comparisons for different cell line databases.

| Dataset | Scenario | Cell Line Feature | AUROC (%) | AUPRC (%) | F1-score (%) |
|---|---|---|---|---|---|
| NCI-ALMANAC | Random | COSMIC | 85.91 | 56.65 | 53.05 |
| | | GDSC | 85.82 | 56.48 | 53.23 |
| | | CCLE (overlap) | 85.06 | 54.01 | 51.32 |
| | | COSMIC (overlap) | 85.26 | 53.99 | 51.21 |
| | CLine | COSMIC | 79.42 | 46.17 | 45.25 |
| | | GDSC | 80.0 | 46.76 | 45.62 |
| | | CCLE (overlap) | 80.54 | 47.08 | 45.64 |
| | | COSMIC (overlap) | 79.74 | 45.59 | 44.67 |
| | DrugComb | COSMIC | 79.75 | 41.71 | 43.43 |
| | | GDSC | 79.08 | 39.59 | 41.75 |
| | | CCLE (overlap) | 78.77 | 37.48 | 40.96 |
| | | COSMIC (overlap) | 78.81 | 37.81 | 41.01 |
| O'Neil | Random | COSMIC | 93.67 | 66.51 | 62.29 |
| | | GDSC | 93.17 | 64.63 | 60.76 |
| | | CCLE (overlap) | 93.41 | 66.24 | 62.54 |
| | | COSMIC (overlap) | 93.44 | 66.36 | 62.9 |
| | CLine | COSMIC | 87.47 | 45.97 | 49.37 |
| | | GDSC | 87.19 | 47.45 | 48.77 |
| | | CCLE (overlap) | 87.32 | 48.97 | 49.95 |
| | | COSMIC (overlap) | 87.75 | 49.08 | 49.61 |
| | DrugComb | COSMIC | 88.34 | 50.02 | 50.0 |
| | | GDSC | 88.43 | 49.5 | 49.75 |
| | | CCLE (overlap) | 88.98 | 52.78 | 52.67 |
| | | COSMIC (overlap) | 89.09 | 53.0 | 52.64 |
